# Congenital diaphragmatic hernia—does the presence of a hernia sac improve outcome? A systematic review of published studies

**DOI:** 10.1007/s00431-020-03779-1

**Published:** 2020-08-17

**Authors:** Arimatias Raitio, Adeline Salim, Paul D. Losty

**Affiliations:** 1 Department of Paediatric Surgery, University of Liverpool, Alder Hey Children’s Hospital NHS Foundation Trust, Eaton Road, Liverpool, L12 2AP UK; 2 Department of Paediatric Surgery, University of Turku and Turku University Hospital, Turku, Finland; 3 Institute of Child Health, University of Liverpool, Liverpool, UK

**Keywords:** CDH, Congenital diaphragmatic hernia, ECMO, Hernia sac, Survival

## Abstract

Early reports have suggested survival benefits associated with a hernia sac in congenital diaphragmatic hernia (CDH). However, these studies have included only small subsets of patients. This systematic review aimed to evaluate differences in outcomes of CDH newborns with and without a hernia sac. PubMed and Embase databases were searched using relevant key terms. Papers were independently reviewed by two authors with final selection approved by the senior author. Original search retrieved 537 papers; the final review included 8 studies (*n* = 837 patients). There were 168 CDH patients (20%) with a hernia sac with an overall survival of 93% vs 73% for CDH newborns without a sac (*p* < 0.001). Twenty-three percent of patients with a CDH sac required diaphragm patch repair vs 44% patients without a sac (*p* < 0.001). Pulmonary hypertension was manifested in 44% of CDH babies with a hernia sac vs 64% without a sac (*p* < 0.001). Three studies compared ECMO requirement: 15% with a hernia sac and 34% without sac, *p* < 0.001.

*Conclusion*: This study shows significant survival benefits in newborns associated with presence of a CDH sac. This may be likely related to these infants having more favourable physiology with less severe pulmonary hypertension and/or smaller anatomical defects requiring primary closure only.

**Table:** 

**What is Known:** *• Early reports have suggested survival benefits associated with a hernia sac in CDH.* *• Previous studies have included only a small number of patients.*	
**What is New:** *• A systematic review of published studies clearly shows that CDH newborns with a hernia sac have better overall survival outcomes and less severe pulmonary hypertension.* *• ECMO utilization and patch repair were also less often required in newborns with a hernia sac.*

**What is Known:**

*• Early reports have suggested survival benefits associated with a hernia sac in CDH.*

*• Previous studies have included only a small number of patients.*

**What is New:**

*• A systematic review of published studies clearly shows that CDH newborns with a hernia sac have better overall survival outcomes and less severe pulmonary hypertension.*

*• ECMO utilization and patch repair were also less often required in newborns with a hernia sac.*

## Introduction

Congenital diaphragmatic hernia (CDH) is the result of diaphragm maldevelopment during foetal life giving rise to pathological herniation of abdominal viscera into the thorax which impacts on normal lung growth associated with pulmonary hypoplasia and hypertension [1]. CDH has an incidence of 1 in 2500 to 3000 live births [2, 3]. Despite current advances in neonatal intensive care, CDH is still associated with an unacceptably high mortality and morbidity [4–6]. Risk factors for poor prognosis currently include liver herniation (‘liver up’), large defect size (type ‘C’ and ‘D’ lesions) and aberrant pulmonary developmental biology [6–10].

In most cases, CDH consists of a direct anatomical defect communicating between the thoracic and abdominal cavities. The presence of a hernia sac with CDH is reported in approximately some 20% of cases [11, 12]. It has been postulated that the presence of a hernia sac may be associated with a better prognosis [11–15]. Although early reports have portrayed survival benefit associated with a hernia sac, this has not been consistent in all studies [16]. Moreover, due to the rarity of a CDH-associated hernia sac, most studies to date have also only described small numbers of patients presenting with a sac anomaly [13, 15, 16].

Against this background, we have therefore undertaken a systematic review study to critically evaluate “Does the presence of a hernia sac in CDH newborns equate with better overall prognosis?”

## Methods

### Identification and selection of studies

A comprehensive search of the published literature in PubMed and Embase databases was performed based on PRISMA (Preferred Reporting Items for Systematic Reviews and Meta-Analyses) guidelines [17]. The following terms were used as keywords: ‘congenital diaphragmatic hernia’ and ‘Bochdalek hernia’ and ‘CDH’ in combination with term ‘sac’. All articles published up to December 31, 2019, were included in the review.

### Inclusion and exclusion criteria

**Fig. 1:**
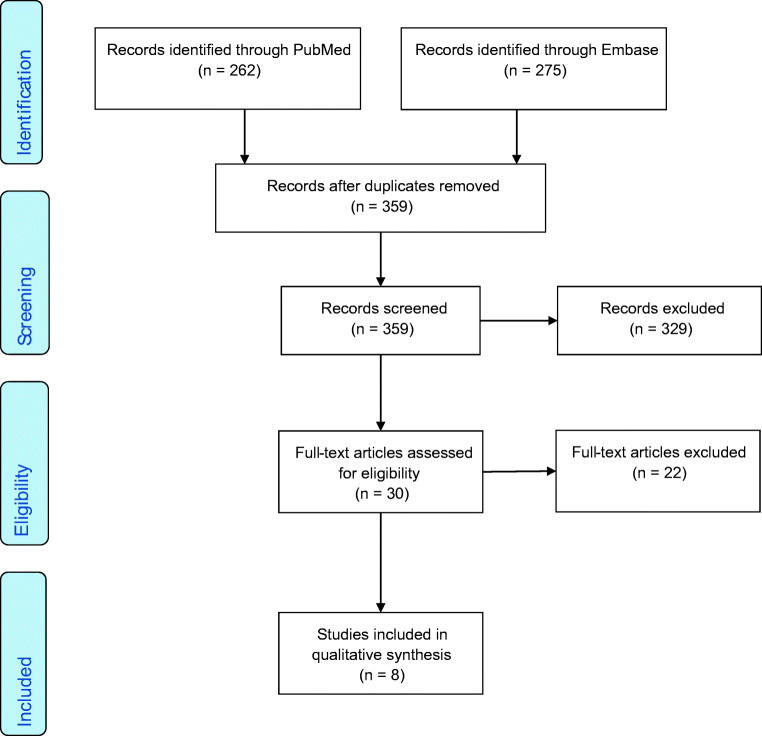
PRISMA study selection flow diagram

This study included all original articles reporting on outcomes of neonatal presentation of congenital diaphragmatic hernia with hernia sac. Non-English language papers and case reports (< 3 patients) were excluded with title and abstract screening. We also excluded studies on late presentations of hernia beyond the neonatal period, acquired defects, other types of diaphragmatic hernia (Morgagni and hiatus/para-oesophageal) and diaphragmatic eventrations (Fig. 1).

### Data extraction and analysis

Identified papers were independently reviewed by two authors with final selection approved by the senior author. The data on presence of a hernia sac, liver herniation, length of hospital stay (LOS), number of days ventilated, pulmonary hypertension, requirement for patch repair, extracorporeal membrane oxygenation (ECMO) and survival was then extracted from the original publications. After summarizing the data, statistical analysis was feasible for all categorical outcome measures. Due to variations in some reporting data by authors on hospital LOS and number of days ventilated, consistent datasets only were consolidated in final review.

### Statistical analysis

Chi-square and Fisher’s exact tests were utilised to analyse categorical variables. A Significance level of *p* ≤ 0.05 (two-tailed) was set. Analyses were performed using JMP Pro, version 13.1.0 for Windows (SAS Institute Inc., Cary, NC, USA).

## Results

The original search identified 537 articles. A total of 359 studies were then evaluated with screening of titles and abstracts after duplicates were excluded. Thirty papers met inclusion criteria in screening and were then selected for full text review. After full text review of 30 articles, eight papers met eligibility criteria and were selected for review (Fig. 1). All selected papers were retrospective single-centre cohort studies. All eligible papers and the extracted data are summarised in Table 1.

**Table 1:** Summary data-articles defining outcomes of CDH newborns with and without a hernia sac

	Case numbers	Survival	Patch repair	Pulmonary hypertension	Liver herniation	ECMO requirement
Zamora et al. (2013)						
With a hernia sac	30 (22%)	25/30 (83%)	6/29 (21%)	5/27 (19%)	23/30 (77%)	3/30 (10%)
Without a sac	107 (78%)	79/107 (74%)	62/97 (64%)	54/102 (53%)	66/107 (62%)	47/107 (44%)
Spaggiari et al. (2013)						
With a hernia sac	18 (26%)	17/18 (94%)	0/18 (0%)	7/18 (39%)	4/18 (22%)	N/A
Without a sac	52 (74%)	35/52 (67%)	4/52 (11%)	33/52 (63%)	11/52 21%)	N/A
Panda et al. (2013)						
With a hernia sac	10 (14%)	9/10 (90%)	0/10 (0%)	3/10 (30%)	2/10 (20%)	N/A
Without a sac	60 (86%)	34/60 (57%)	6/60 (10%)	37/60 (62%)	14/60 (23%)	N/A
Grizelj et al. (2017)						
With a hernia sac	7 (24%)	7/7 (100%)	1/7 (14%)	5/7 (71%)	7/7 (100%)	N/A
Without a sac	22 (76%)	7/22 (32%)	5/22 (23%)	17/22 (81%)	22/22 (100%)	N/A
Bouchgoul et al. (2018)						
With a hernia sac	17 (24%)	17/17 (100%)	2/17 (12%)	11/17 (65%)	6/17 (35%)	N/A
Without a sac	55 (76%)	35/55 (64%)	24/48 (50%)	38/55 (69%)	27/55 (49%)	N/A
Aydin et al. (2019)						
With a hernia sac	26 (14%)	25/26 (96%)	7/26 (27%)	N/A	18/26 (69%)	4/26 (15%)
Without a sac	162 (86%)	130/162 (80%)	58/162 (36%)	N/A	108/162 (67%)	57/162 (35%)
Levesque et al. (2019)						
With a hernia sac	14 (20%)	14/14 (100%)	6/14 (43%)	5/14 (36%)	6/14 (43%)	N/A
Without a sac	57 (80%)	54/57 (95%)	30/57 (53%)	28/57 (54%)	17/57 (30%)	N/A
Oliver et al. (2019)						
With a hernia sac	46 (23%)	N/A	16/41 (39%)	23/41 (56%)	N/A	8/41 (20%)
Without a sac	154 (77%)	N/A	90/130 (69%)	98/130 (75%)	N/A	33/130 (25%)

*N/A* data not available

In total, there were 837 patients with congenital diaphragmatic hernia including 168 patients (20%) with a hernia sac. Overall survival was significantly better in the patient groups with a hernia sac (93% vs 73%, *p* < 0.001). Requirement for patch repair of the defect was significantly more common among those without a hernia sac (44% vs 23%, *p* < 0.001). Pulmonary hypertension was manifested more often among CDH babies without a hernia sac (64% vs 44%, *p* < 0.001). Three studies compared the requirement for ECMO. Absence of a hernia sac was associated with more frequent ECMO utilization (34% vs 15%, *p* < 0.001). There were no significant difference(s) observed in the presence of liver herniation (‘liver up’) between the groups though ‘liver up’ was more common in newborns with a hernia sac (54% vs 49%, *p* = 0.34) (Table 2).

**Table 2:** Comparison of outcomes between CDH newborns with and without a hernia sac

	Survival	Patch repair	Pulmonary hypertension	Liver herniation	ECMO requirement
With hernia sac *n* = 168 (20%)	114/122 (93%)	38/162 (23%)	59/134 (44%)	66/122 (54%)	15/97 (15%)
Without sac *n* = 669 (80%)	374/515 (73%)	279/628 (44%)	305/478 (64%)	243/493 (49%)	137/399 (34%)
*P* value	< 0.001	< 0.001	< 0.001	0.34	< 0.001

All six studies which analysed hospital/neonatal intensive care LOS reported shorter LOS in the CDH group with a hernia sac although this difference was only statistically significant in three of those six studies. Seven studies examined number of ventilation days and although all reported shorter duration among CDH newborns with a hernia sac, statistical significance was observed here in only two studies.

## Discussion

This systematic review study has clearly shown that CDH newborns with a hernia sac have better overall survival outcomes and less severe pulmonary hypertension. Moreover, ECMO utilization and patch repair were equally less often required in newborns having a hernia sac noted at the timing of the operative repair of the diaphragm defect.

Some early reports have made effort to show that the presence of hernia sac may be associated with better survival [11, 14, 15, 18]. However, difference(s) were not statistically significant in all these studies [16]. As pulmonary hypoplasia is a significant risk factor for mortality in newborns with CDH [10] and the presence of hernia sac is now reportedly associated with higher total foetal lung volumes by foetal medicine centres [11, 12], we postulate that the benefits in survival we now report from a systematic review of ‘high quality’ published studies here may be potentially linked with higher total lung volumes also preserved in the postnatal period in CDH infants surviving with a hernia sac.

Pulmonary hypertension is a key factor linked with mortality and morbidity in CDH [8, 19]. Only one study has reported a potential association between pulmonary hypertension and presence of a hernia sac in CDH [12]. The combined data we have now analysed clearly show a significant difference in frequency of pulmonary hypertensive episodes in CDH babies with and without a hernia sac. The better physiological outcomes observed in CDH newborns with a hernia sac are intriguing.

Prosthetic patch repair was required much less often in infants studied here with a hernia sac. Similar, independent finding(s) were reported in three of eight studies we have reviewed [12, 14, 20]. These findings strongly suggest smaller defect size (‘A’ and ‘B’ category type defects) associated with presence of a hernia sac. Interestingly, we observed that CDH cases with hernia sac were more often associated with liver herniation though the difference(s) were not statistically significant vs infants with no hernia sac. Intrathoracic liver herniation is considered to be a strong prognostic marker in foetal CDH and associated with poor outcome(s) [7, 10, 21]. In our current study, we found that more than half of the CDH cases we analysed with a hernia sac had reportedly liver herniation and yet a very high survival rate of almost 93%. It therefore seems evident that the presence of a hernia sac in some way may be a ‘protective’ native biological barrier favouring preservation of lung growth in affected foetuses. Identification of a hernia sac ‘in utero’ with foetal MRI imaging may potentially serve as a new prognostic marker for better outcome(s).

Requirement for ECMO was reported in three studies we analysed [12, 20, 21] with one study clearly showing statistically significant difference in outcome [12]. The combined data from all three studies established that ECMO was less often required in infants with a hernia sac. Although we were unable from the current publications to fully analyse data on hospital LOS and number of days ventilated, the emerging evidence suggests that the presence of CDH hernia sac is associated with shorter LOS [12, 20, 21], and also fewer hospital days requiring ventilation [12, 16]. These findings are most likely reflective of more favourable physiology with better lung growth preserved in infants (see earlier) also with reduced rates of pulmonary hypertension.

Some limitation(s) of the current systematic review relate to variations in methods of data reporting from the published studies. Hence, we were unable to fully analyse metrics on hospital LOS and total number of days ventilated. As all included papers were single-centre studies, we believe that the study authors reporting of pulmonary hypertension was standardised in individual units minimizing centre bias. Finally, all included studies analysed were retrospective cohort populations.

This study has demonstrated that the presence of a hernia sac in CDH is associated with survival benefits. These finding(s) may be likely related to CDH newborns having more favourable physiology such as reduced rates of pulmonary hypertension and/or smaller diaphragm defects (‘A’ and ‘B’ category) which are amenable to primary closure and repair.

## Abbreviations

CDH: Congenital diaphragmatic hernia
ECMO: Extracorporeal membrane oxygenation
LOS: Length of stay
